# Classification of Domain Movements in Proteins Using Dynamic Contact Graphs

**DOI:** 10.1371/journal.pone.0081224

**Published:** 2013-11-18

**Authors:** Daniel Taylor, Gavin Cawley, Steven Hayward

**Affiliations:** D'Arcy Thompson Centre for Computational Biology, School of Computing Sciences, University of East Anglia, Norwich, United Kingdom; Jacobs University Bremen, Germany

## Abstract

A new method for the classification of domain movements in proteins is described and applied to 1822 pairs of structures from the Protein Data Bank that represent a domain movement in two-domain proteins. The method is based on changes in contacts between residues from the two domains in moving from one conformation to the other. We argue that there are five types of elemental contact changes and that these relate to five model domain movements called: “free”, “open-closed”, “anchored”, “sliding-twist”, and “see-saw.” A directed graph is introduced called the “Dynamic Contact Graph” which represents the contact changes in a domain movement. In many cases a graph, or part of a graph, provides a clear visual metaphor for the movement it represents and is a motif that can be easily recognised. The Dynamic Contact Graphs are often comprised of disconnected subgraphs indicating independent regions which may play different roles in the domain movement. The Dynamic Contact Graph for each domain movement is decomposed into elemental Dynamic Contact Graphs, those that represent elemental contact changes, allowing us to count the number of instances of each type of elemental contact change in the domain movement. This naturally leads to sixteen classes into which the 1822 domain movements are classified.

## Introduction

From a structural perspective domains in proteins can be regarded as quasi-globular regions. The connections between domains allow their relative movement and consequently domain movements are often engaged in protein function [1], [2]. The Protein Data Bank (PDB) [3] is a rich source of information on protein domain movements as for a number of proteins, multiple structures have been deposited. Differences in structure may be due to functional changes in state as occurs upon the binding of a natural ligand, but may also be due to differences in the experimental conditions under which the structures were solved, or could be due to natural or engineered mutations. The implied movements between multiple structures of certain proteins deposited in the PDB invite a computational biology approach in order to understand principles and causes of protein conformational change. For domain proteins there have been a number of such studies. As understanding in biology often follows classification of experimental findings some of these studies have attempted to classify the implied movements in domain proteins.

In an influential review of protein domain movements using structures from the PDB, Gerstein et al. [4] saw two main types: predominantly hinge and predominantly shear. Following this study the DataBase of Macromolecular Movements (DBMM) appeared online with further examples [5]. A number of other large-scale studies have been made using structures from the PDB each approaching the problem from a different perspective. A study of movements in enzymes upon substrate binding reported that they are generally small [6], although another study has shown that the extent of movement may depend on the actual reaction mechanism [7]. A study based on the DynDom program [8], [9] for the analysis of domain movements in proteins considered structural features of hinge-bending regions [10] and the application of the same program to create a Non-redundant DataBase of Protein Domain Movements (NRDPDM) showed that protein domain movements are very controlled in the sense that many different structures from the same family represent the same domain movement [11]. The “Database of Ligand-Induced Domain Movements in Enzymes,” [12] which is a subset of the NRDPDM, categorised domain movements in 203 enzymes based on whether a ligand “spans” the two domains or not and whether the ligand has caused compaction of the proteins upon binding. A more general approach has been taken to produce the Protein Structural Change DataBase (PSCDB) [13], [14] where 839 protein movements between liganded and unliganded structures have been classified into seven categories: “coupled domain motion”, “independent domain motion”, “coupled local motion”, “independent local motion”, “burying ligand motion”, “no significant motion”, and “other type of motion”. Related to these studies is another large scale study which considered 521 structural pairs with the conformational change apparently induced by ligand binding [15]. Although this study did not classify domain movements it did consider the predictability of domain movements from the ligand-free form. Another way to approach the subject of domain movements in proteins is to consider the energetics of the process. Sinha et al. [16] showed that for a number of domain proteins the nonpolar buried surface area in the open state matches or exceeds the nonpolar buried surface area in the closed state.

The method presented here is based on changes in interdomain residue contacts that occur in the domain movement. The advantage of such a method is that it is relatively simple to implement but has a connection to methods based on calculating interaction energies. Key to the analysis is the concept of the “Dynamic Contact Graph” (DCG). Each domain movement has an associated DCG. Using graphs has three benefits: they provide a visual metaphor for the movement they represent; they provide motifs for some basic domain movements that are instantly recognisable; the well-developed algorithms of graph theory can be used to evaluate features of interest. The analysis is developed in terms of “elemental” DCGs which represent elemental contact-changes. These elemental contact-changes can, under certain assumptions, be associated with “model” domain movements. We count the number of elemental DCGs any general DCG comprises which naturally leads to sixteen different categories into which the domain movements are classified. The results are presented at a website.

## Methods

### Database

The basic data are the 2035 unique domain movements from the NRDPDM [11]. The domain movements were determined by the DynDom program[8], [9]. These unique movements come from 1578 families which means that some domain movements are from the same family. Individual cases from this dataset are available to browse at http://www.cmp.uea.ac.uk/dyndom. In order to simplify the analysis only those cases with two domains were used. Of the 2035 cases, 1822 are two-domain proteins. The two domains in each protein will be referred to as “domain A” and “domain B” below.

### Residue contact definition

“Contact” between residue i and residue j means any heavy atom of residue i is within 4 Å of any heavy atom of residue j. However, before the set of pair-wise contacts between residues in each domain and for each conformation is determined, residues at the boundaries of the domains annotated by DynDom as bending regions were removed as were residues close to the interdomain screw axis (any heavy atom of the residue within 5.5 Å of the axis). The reason for this is that they would be expected to have maintained contacts (see below) irrespective of the nature of the domain movement.

### Elemental contact-changes and model domain movements

Let (a_1_,b_1_) denote a “residue contact pair”, where a_1_ is the residue number of a residue in domain A, and b_1_ is the residue number of a residue in domain B, that make contact in conformation 1. Similarly let (a_2_,b_2_) represent a residue contact pair in conformation 2. By considering *at most* a single residue contact pair between the domains in *either* conformation there are five “elemental contact-change” scenarios (where below () indicates no contact exists):

- “**no-contact**”: (a_1_,b_1_)  = () and (a_2_,b_2_)  = ().
- “**new**”: either (a_1_,b_1_) ≠() and (a_2_,b_2_)  = () or (a_1_,b_1_)  = () and (a_2_,b_2_) ≠().
- “**maintained**”: (a_1_,b_1_) ≠() and (a_2_,b_2_) ≠() where a_1_ = a_2_ and b_1_ = b_2_.
- “**exchanged-partner**”: (a_1_,b_1_) ≠() and (a_2_,b_2_) ≠() where (a_1_ = a_2_ and b_1_≠b_2_) or (a_1_≠a_2_ and b_1_ = b_2_).
- “**exchanged-pair**”: (a_1_,b_1_) ≠() and (a_2_,b_2_) ≠() where a_1_≠a_2_ and b_1_≠b_2_.

The contact-changes can be associated with five “model” domain movements assuming the following idealisation.

- The domains have a spherical shape and are perfectly rigid.
- There is only one residue from each domain at a contact point.
- The relative movement of the domains is a rotation about a hinge axis passing through an interdomain linker region which is short in comparison to the size of the domains.

**Figure 1:**
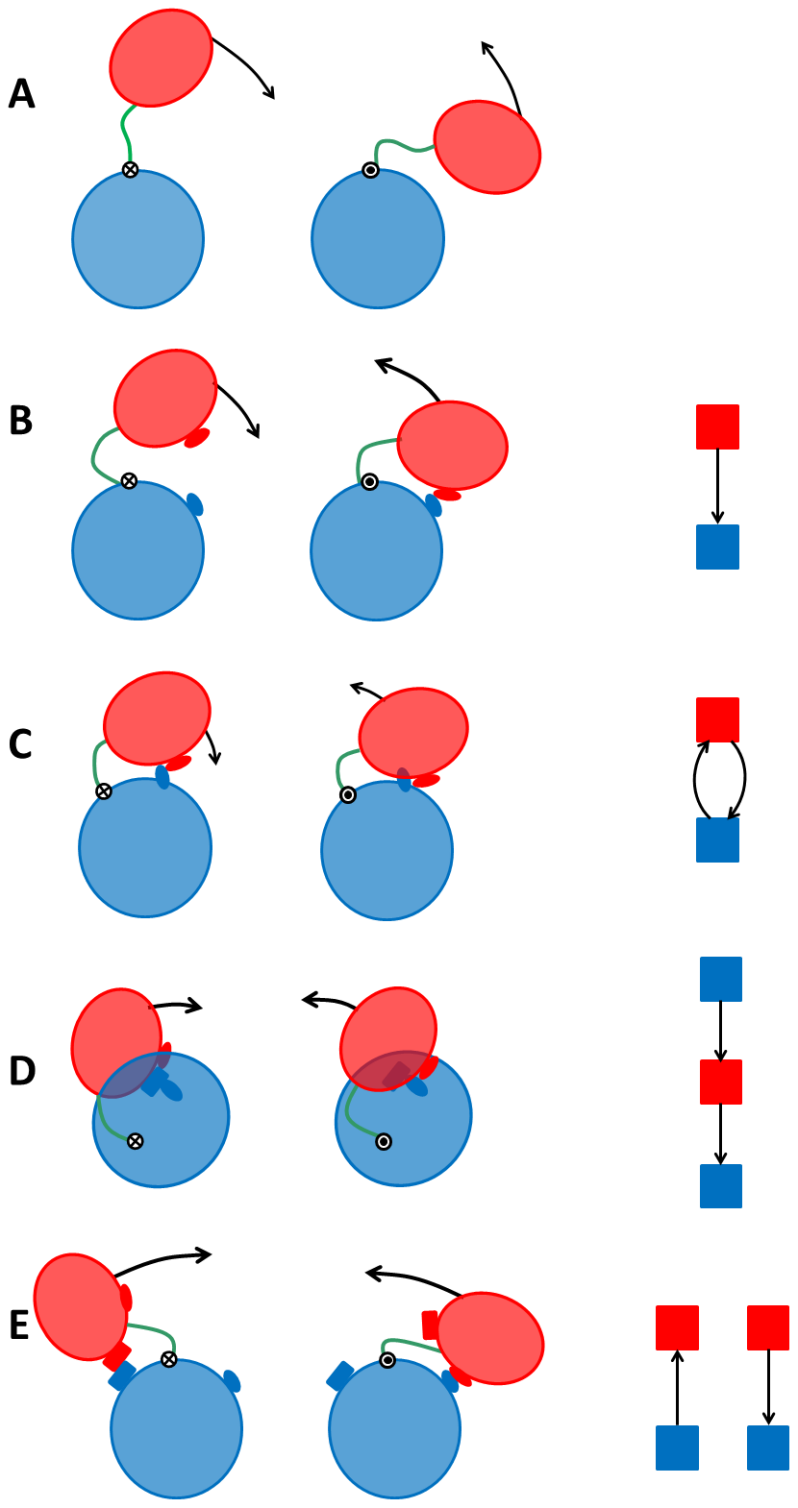
The five model domain movements and their corresponding elemental DCGs. Conformation 1 is on the left and conformation 2 on the right with domain A in blue and domain B in red. (A) The “no contact” contact-change implies that the domains are “free” to move. The graph is empty in this case. (B) The “new” contact-change implies an “open-closed” domain movement. In this case the elemental DCG shows a contact between the two domains in conformation 2 as indicated by the edge-arrow pointing from domain B to domain A. (C) The “maintained” case implies the domains are “anchored” and the associated DCG is a doubly-linked motif. (D) The “exchange-partner” contact-change is where a residue, here on domain B, makes a contact with a residue on domain A in conformation 1 and a contact with a different residue on domain A in conformation 2. This implies a model “sliding-twist” movement whereby domain B slides on the surface provided by domain A. The elemental DCG provides a visual metaphor for this movement with arrows indicating a movement away the contacting residue on domain A in conformation 1 (upper blue node) towards the contacting residue on domain A in conformation 2 (lower blue node). (E) The “exchanged-pair” contact-change and its associated model “see-saw” movement. The DCG clearly depicts this kind of see-saw movement.

The “no contact” case implies the domains remain separated and can move freely. This case we call “*free*”. The “new” case implies the domains move from a contacting to non-contacting conformation (or vice-versa) suggesting a rotation about a hinge axis perpendicular to the line joining the centres of mass of the domains, defined previously as a “closure” motion[17]. This is called an “*open-closed*” movement. The “maintained” case means the domains cannot move (given that we exclude the hinge region which would otherwise be designated as maintained region) implying the domains remain “*anchored*”. For the “exchanged-partner” case we have the same residue from one domain making a contact in both conformations but with different residues on the other domain. This implies one domain sliding over the other and is easiest to imagine occurring by a relative twist of the domains. Consider the hinge axis passing through the centre of mass of domain A, with the centre of mass of domain B slightly shifted from the hinge axis, i.e. predominantly a twist motion [17]. If contact occurs between the two domains then the contact point (residue) on domain B will trace out a circle on domain A. So, residue B will contact two different points (residues) on domain A in a movement. We call this movement a “*sliding-twist*”. For the “exchanged-pair” case, the two residues making contact in one conformation are not involved in making contact in the other conformation again implying a movement with the hinge axis perpendicular to the line joining the centres of mass. The movement would break the contact on one side of the domains and rotation continues until contact is made on the other side of the domains. This is commonly known as a “*see-saw*” motion which has already been seen to occur in lactoferrin [18]. More realistic interpretations of these five model domain movements with non-spherical domains and residues of finite size are illustrated in Figure 1.

The association of these elemental contact-changes with the model domain movements is based on consideration of the simplest, most plausible domain movement to reproduce the elemental contact-change in an idealised system. In reality even in those cases where only one type of elemental contact-change occurs, the movement might not resemble the corresponding model domain movement as domains are not perfectly rigid and often have complex interfaces. The extent to which real domain movements conform to these idealised movements is something to be determined.

### Dynamic Contact Graphs

Here we introduce Dynamic Contact Graphs (DCGs). Let {(a_1i_,b_1i_)}, i = 1,N_1_ denote the set of residue contact pairs in conformation 1 and {(a_2i_,b_2i_)}, i = 1,N_2_ the corresponding set for conformation 2.

Each node of the graph represents a residue of which there are two types: those in domain A and those in domain B. An edge exists when there is a contact between a residue in domain A and a residue in domain B, i.e. when they appear in one of the sets above. The key feature of a DCG is that it is directed. For contacts in conformation 1 the direction associated with an edge is from the residue (node) in domain A to the residue (node) in domain B (call this an AB edge). This could be written as a_1i_→b_1i_. For contacts in conformation 2 the direction is from the residue (node) in domain B to the residue (node) in domain A (call this a BA edge). This could be written as a_2i_←b_2i_. Figure 1 shows the “elemental DCGs” for the five model domain movements.

In general a domain movement may combine these elemental contact-changes and have a complex graph structure.

We make full use of Matlab (version 8.0.0.783 (R2012b)) and in particular the Bioinformatics Toolbox “biograph” function to create a “biograph” object, a data structure for directed graphs. This enabled us to use associated methods to analyse and view the DCGs.

## Results

Information on each domain movement can be found at our website. Each domain movement has its own webpage on which its DCG is shown. However, 413 domain movements have no contacts in both conformations (apart from at the removed hinge regions). For these the DCG is empty. These domain movements are assigned to the “no contact” class which implies a free movement of the domains.

**Figure 2:**
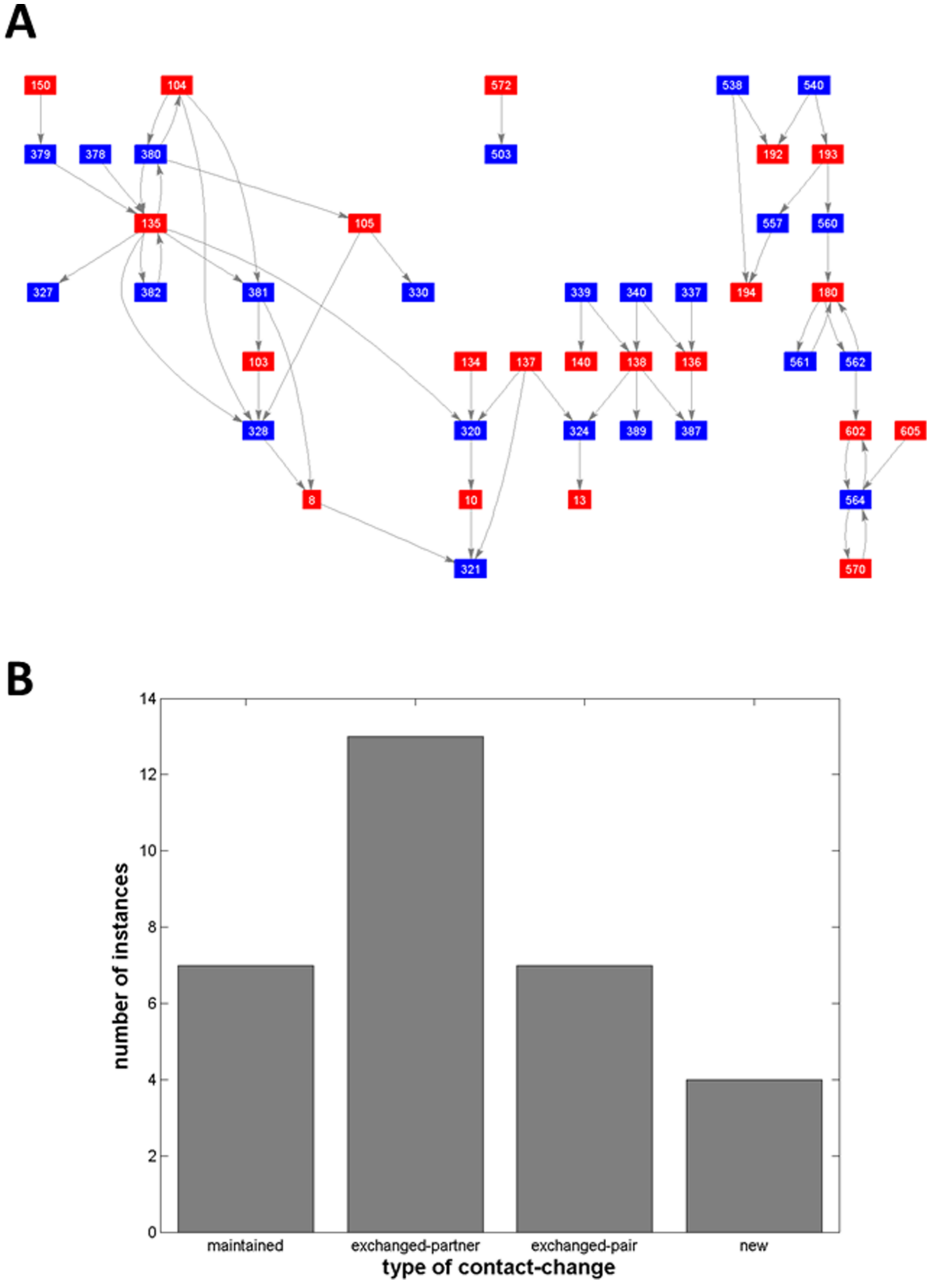
DCG and bar chart for DNA topoisomerase III. (A) DCG for DNA topoisomerase III for the movement between structural pair: 1I7D, chain A, and 1D6M, chain A. (B) Decomposition of the DCG determines the number of instances in each of the four types of elemental contact-changes, “maintained”, “exchanged-partner”, “exchanged-pair” and “new”, which are displayed in a bar chart.

The remaining 1409 domain movements each have a DCG. An illustrative example from a DNA topoisomerase III is shown in Figure 2. Our aim is to process each DCG in order to count how many of each of the four elemental contact-changes are contained within it (we ignore no contact which applies to all the residues not contained in the graph and is only interesting when all residues in the protein are in this category). The distribution of the number of instances of each of the four elemental contact-changes in each DCG will allow us to classify the domain movements.

### Decomposing DCGs into the elemental contact-changes - principles

As can be seen in Figure 2, DCGs are not necessarily connected. A disconnected graph means that residues in one subgraph do not make contact with any residues from another disconnected subgraph in either conformation, indicating independent regions that are possibly playing a different role in the domain movement. We use the Matlab Bioinformatics Toolbox's “biograph” object method “conncomp” to count the number of disconnected subgraphs for all DCGs. This information is presented on the webpage of each domain movement.

**Figure 3:**
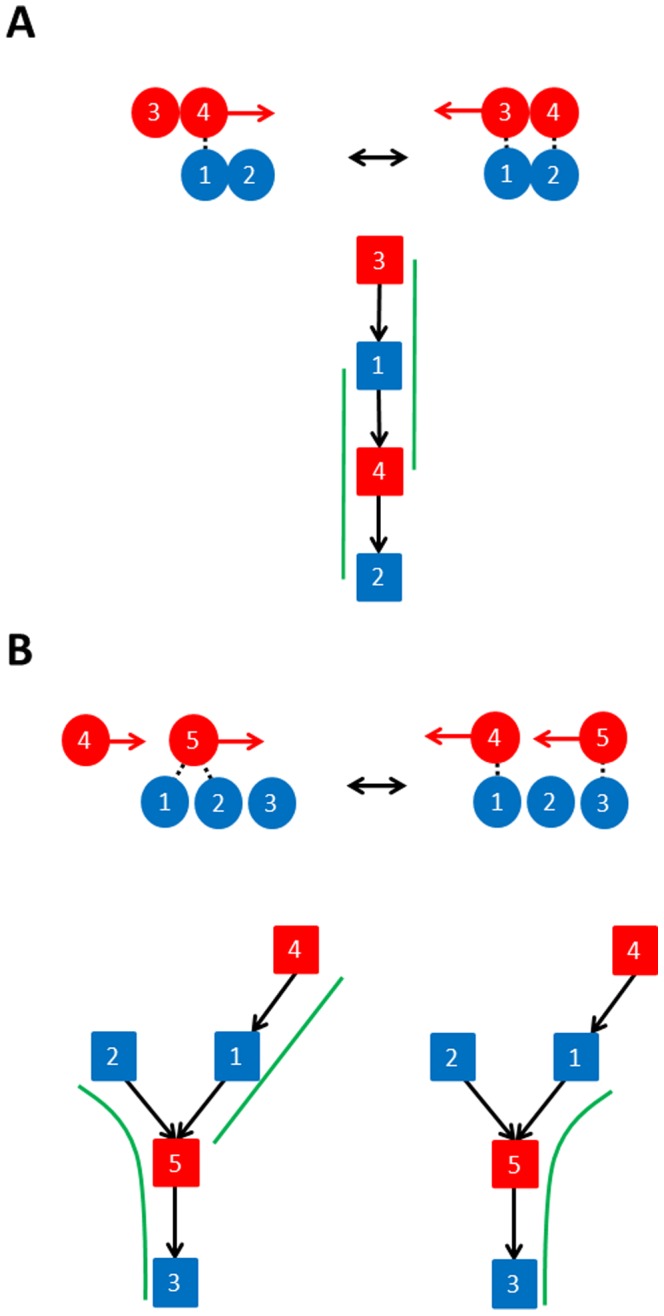
Illustrations of the ambiguity in decomposing a DCG into the elemental “exchange-partner” DCGs. Filled circles indicate residues, those coloured blue are from domain A and those coloured red from domain B. A contact is indicated by a broken line. (A) Top: residues 3 and 4 on domain B slide on residues 1 and 2 on domain A. This can be interpreted as either residue 4 sliding on the surface provided by 1 and 2 or residue 1 sliding on the surface provided by 3 and 4. Bottom: for the associated DCG the elemental “exchange-partner” DCGs are indicated by the green lines but only one can be selected as they should not overlap. (B) Top: residues 4 and 5 on domain B slide on residues 1, 2 and 3 on domain A. Bottom: there are two decomposition possibilities of the DCG indicated by the green lines, one with two non-overlapping elemental “exchange-partner” DCGs (left), and the other with one non-overlapping elemental “exchange-partner” DCG (right).

Our aim is to count the number of contact-changes of each type for each domain movement. This is equivalent to decomposing a DCG into the four elemental DCGs shown in Figure 1. Identifying a contact change implies that a pair of contacts in one conformation have to be associated with a pair of contacts (or indeed lost contacts) in the other conformation. Identifying and counting maintained contact-changes (which appear as double links in the graph) is an unambiguous process. Let N_maint_ represent the number of maintained-changes. For the DNA topoisomerase III shown in Figure 2 N_maint_ = 7. Counting exchanged-partner contact-changes is not unambiguous as illustrated in Figure 3. In Figure 3A there is a single contact between residues 1 and 4 in conformation 1, but after a sliding movement there are two contacts in conformation 2. The ambiguity lies in whether it is residue 1 that exchanges contact partner 4 with 3, or whether it is residue 4 that exchanges contact partner 1 with 2. In the DCG this is equivalent to identifying the elemental DCGs for an exchanged-partner contact-change which is a triplet (three nodes connected by two edges with the same direction). In this example we can select the triplet 3-1-4 or the triplet 1-4-2. Note that we cannot count both as we are counting types of contact-changes and counting both would mean that the 1-4 contact is counted twice. If we select the triplet 3-1-4 then the new contact is 2-4; if we select the triplet 1-4-2 then the new contact is 1-3 and in the absence of any further information both are valid. In practice only one will be selected (see below). This example shows that for exchanged-partner contact-changes we should select only non-overlapping triplets in a DCG.

Figure 3B illustrates another example where there are two possible solutions. One solution has two exchanged-partner contact-changes: residue 1 (in domain A) slides on the surface of residues 4 and 5 (in domain B), and residue 5 (in domain B) slides on the surface of residues 2 and 3 (in domain A). The other solution gives just one exchanged-partner contact-change: residue 5 (in domain B) slides on the surface provided by residues 1 and 3 (in domain A). If we choose the latter then the interactions between residues 2 and 5 in conformation 1 and 1 and 4 in conformation 2 would be assigned to an exchanged-pair contact-change, indicating a possible see-saw movement. In terms of the DCG one can easily see that there are two possible ways to fit non-overlapping triplets in this graph, one gives one triplet, the other, two triplets. How do we in the absence of any other information decide which one to select? Although both are possible, it is more likely that given some of the residues are in an exchange-partner contact-change indicating a sliding movement then all residues would be sliding and therefore in an exchanged-partner contact-change. Therefore we should maximise the number of exchanged-partner contact-changes in a graph. An alternative argument would be that we should maximise the number of associated contact pairings in a graph (in an exchanged-partner contact-change two contact pairs one from each conformation are associated via the residue that appears in both) before pairing off contact pairs to the exchanged-pair contact-changes for which there is no association.

The problem of identifying exchanged-partner contact-changes is therefore equivalent to finding the maximum number of non-overlapping triplets in the DCG.

Once the maintained and exchanged-partner contact-changes have been assigned the exchanged-pair and new contact-changes are assigned as detailed below.

### Decomposing DCGs into the elemental contact-changes - practice

The first step counts the number of maintained contact-changes in a DCG and then creates a new DCG that has no double links. The maximum number of non-overlapping triplets in the resulting graph was then determined as follows. First all possible triplets (overlapping and non-overlapping) were determined. A new (undirected) graph was then created which had a node (vertex) for each triplet and an edge between any two nodes with triplets that overlap. An exhaustive search was implemented to find the maximum number of non-overlapping triplets. The algorithm involved selecting a node, removing those nodes connected with it by a single edge and repeating this process until no nodes remain. The selected nodes give a set of non-overlapping triplets. This recursive program is given here in pseudo-code:

Input: A graph with vertices (nodes, representing triplets) ordered, *V* = *v_1_,v_2_,v_3_,.. ,v_n_* and a set of edges *E* (an edge existing if the two vertices represent triplets that overlap).

Output: A list of vertices, *W_max_* , with the maximum number of vertices, *N_max_* , none of which are connected by a single edge.

*N_max_ = 0*

*W_max_* = {}

*W* = {}

add *v_1_toW*

*w* = *v_1_*

*V’* = *V*

*unconnected* ( *w,V’,E,W,W_max_,N_max_){*

if (| *V’* | = 0){

if (| *W* |> *N_max_){*

*W_max_ = W*

*N_max_ = |W|*

}

return *W_max_,N_max_*

# terminate branch in search tree if it cannot

# exceed *N_max_*

}elseif (| *V’* |+| *W* |< = *N_max_* ){

return

}

while (there is an edge (*w*,*v_j_*)ϵ*E*) {

remove *v_j_* from *V’*

}

remove *w* from *V’*

add *v_i_* to *W* # *v_i_* appears first in *V’*

*w* = *v_i_*

# recursive call to unconnected

*unconnected*(*w,V’,E,W,W_max_*,*N_max_*)

}

For twelve DCGs this exhaustive search was too slow and was replaced by a related random search (Repeat the following N times: randomly select a vertex *w*, add to *W*; remove vertices with an edge connecting to *w*; continue first two steps until exhaustion of vertices. Then search amongst the N *W* recorded for each repetition for *N_max_* and *W_max_*). This random search found the same value of *N_max_* determined by the exhaustive search in all 1397 DCGs that could be search exhaustively. N_exchpart_, the number of exchanged-partner contact-changes is set equal to *N_max_*.

The maximum number of non-overlapping triplets is not a unique set but only one is delivered by the exhaustive search given above. For the purpose of this study it does not matter which set we select as we are interested only in the number of each type of elemental contact-change.

A DCG with maintained and exchanged-partner contact-changes removed comprises disconnected two-node subgraphs. Each subgraph has a single AB edge for conformation 1 or a single BA edge for conformation 2 and these are paired off to count the number of exchanged-pair contact-changes. Let n_1_ be the number of remaining conformation 1 contacts after the maintained and the exchanged-partner contact-changes have been removed, and likewise n_2_ be the number of remaining conformation 2 contacts. The number of exchanged-pair contact-changes was taken to be N_exchpair_  = min(n_1_,n_2_). In a DCG with maintained, exchanged-partner and exchanged-pair contact-changes removed there are only two-node subgraphs of one type left, either AB or BA. These represent the new contact-changes. The number of new contact-changes, N_new_, is then given by N_new_  = n_1_- N_exchpair_ or N_new_  = n_2_- N_exchpair_, the former if n_1_≥n_2_, the latter if n_2_>n_1_.

For the example in Figure 3B this process would result in N_maint_ = 0, N_exchpart_  = 2, N_exchpair_  = 0 and N_new_ = 0. For the less trivial case of DNA topoisomerase III shown in Figure 2, N_maint_ = 7, N_exchpart_  = 13, N_exchpair_  = 7 and N_new_ = 4.

### Classifying domain movements

We classify domain movements according to which of the contact-change categories are non-empty or empty. There are five types of contact-change, but given that for all domain movements there are always residues that do not make interdomain contacts in both conformations, the no contact-change case is redundant. The only interesting case is when all residues are in this category but this case is covered when the number of contact-changes in all the other categories is zero. Therefore we need only consider the remaining four contact-change categories.

Each of the four categories can be empty or non-empty meaning there are sixteen (2^4^) different classes. The no-contact class is when all four categories are empty. There are four “pure” classes, when only one category is non-empty, the other three being empty, e.g. “pure new” has N_maint_ = 0, N_exchpart_  = 0, N_exchpair_  = 0, N_new_≥1. There are six classes when two categories are non-empty and two empty, e.g. “combined maintained, new” has N_maint_≥1, N_exchpart_  = 0, N_exchpair_  = 0, N_new_≥1. There are four classes when three categories are non-empty and one empty, e.g. “combined exchanged-pair, exchanged-partner, new” has N_maint_ = 0, N_exchpart_ ≥1, N_exchpair_ ≥1, N_new_≥1. Finally, there is one class when all four categories are non-empty. These classes are given in Table 1 alongside the number of domain movements in each class.

**Table 1:** Numbers in each class.

Class	N° of examples
Pure no contacts	412
Pure maintained	56
Pure exchanged-partner	3
Pure exchanged-pair	9
Pure new	376
Combined maintained, exchanged-partner	10
Combined maintained, exchanged-pair	44
Combined maintained, new	225
Combined exchanged-partner, exchanged-pair	1
Combined exchanged-partner, new	34
Combined exchanged-pair, new	78
Combined maintained, exchanged-partner, exchanged-pair	35
Combined maintained, exchanged-partner, new	126
Combined maintained, exchanged-pair, new	137
Combined exchanged-partner, exchanged-pair, new	53
Combined all	223

It is interesting that there are so many examples of domain movements where no contacts are made between the domains (except at the hinge bending sites) in both conformations. Some of these may be due to domain linkers that act as rigid spacers between the domains to prevent unfavourable interdomain interactions during folding [19].

In terms of the total number of contact-change types across the whole set, there are 6810 new, 6087 maintained, 1448 exchanged-pair and 1150 exchanged-partner contact-changes.

### Website for domain movement classification

We have produced a website where the domain movements are organised according to class (see http://www.cmp.uea.ac.uk/dyndom/class16). Each class comprises a list of protein names together with a pair of PDB accession codes and chain identifiers that specify the domain movement. The link provided takes one to a page where the DCG and a bar chart for the distribution of the number of instances in each of the four elemental contact-change categories are shown (see Figure 2). The number of independent regions is also given. The molecular graphics applet, Jmol (http://jmol.sourceforge.net/), is used to display the movement and to indicate the residues that make contact in each conformation. There is also a link to the corresponding DynDom page for that domain movement which gives details on the residues comprising the domains, the location of the hinge axis, the hinge-bending residues, the angle of rotation, percentage closure, as well as many other details, and a downloadable script for viewing the movement. A link to the DynDom family page is also provided which gives a conformational analysis of closely related structures and their domain movements [11].

### Real domain movements and the model domain movements

In the Methods section we proposed an association between the elemental contact-changes and model domain movements. This association requires the domains and domain movements fulfil a set of conditions that are unlikely to be satisfied in real cases. Amongst others these conditions require the domains to be perfectly rigid and be convex in shape. It is clear from our results that many domain movements combine the four different types of elemental contact-changes suggesting immediately that the model domain movements are not appropriate for these cases. Even in the “pure” cases the model domain movements may not provide an appropriate description of the movement.

The model domain movement associated with the no contact set is the free domain movement implying the domains are free to move relative to each other but never make contact. This fact cannot be determined from just two structures and therefore we are unable to judge from our data whether the domains are free.

The pure new class implies the open-closed model movement; that is a movement that is predominantly a closure motion[17]. We can see an example that conforms to this model in Lysine-, Arginine-, Ornithine-binding (LAO) Protein (search for PDB accession codes 2LAO and 1LST on the main webpage). The protein has a well-defined hinge axis that brings the two rather globular domains together in a motion that is 99% closure. However, there are many examples in this class that do not conform to this model. An example can be seen in the domain movement in the human cellular receptor for Epstein-Barr virus (PDB codes 1GHQ and 1LY2) where contact is established via a twist motion (6.7% closure).

For the pure maintained class the corresponding domain movement is anchored and indeed only 12.5% of this class have rotations of more than 15° compared to 74.2% for the pure new indicating that maintained contacts do restrict rotation. However, because this group have small rotations domain demarcation becomes more subject to noise and many of these cases are due to only a slight difference in the rotational properties of the residues that maintain contact.

There are only three examples in the pure exchanged-partner class none of which are like the expected sliding twist model domain movement. The example of DnaA, a chromosomal replication initiator protein (PDB codes: 1L8Q and 2HCB), shows that an exchanged-partner contact-change can occur without a sliding twist movement if the interdomain screw axis is remote from the interdomain region, i.e. it violates one of the conditions for a model domain movement. A sliding twist movement is seen, however, in an immunoglobulin protein in the combined exchanged-partner, new class (PDB codes: 1E4K and 2IWG) where the domain movement is predominantly a twist (37% closure).

Finally in the pure exchanged-pair class which is associated with the see-saw model domain movement, six out of the nine examples would conform to a see-saw movement in that one can find a plane that the interdomain screw axis lies in and for which the contacts in the two conformations occur on either side of this plane. An example can be see for a histidine kinase (PDB codes: 1B3Q and 2CH4) which undergoes a clear see-saw movement with the domains rotating through 126°. An example that would not seem to be like a see-saw movement can be see for a lytic transglycosylase (PDB codes: 2G6G and 2G5D) where the non-globular shape of the domains and their location in relation to the hinge axis allows an exchanged-pair contact-change to occur via a non see-saw-like movement.

## Discussion

We have used a contact analysis to help classify domain movements in proteins. The approach introduced here is based on identifying five types of elemental contact-changes. A real domain movement will comprise these elemental contact-changes but decomposing contact-changes in a real domain movement into the elemental contact-changes is non-trivial. A solution to this problem was found by encoding the contact-changes in a DCG and decomposing it in terms of the elemental DCGs which represent the elemental contact-changes. This allowed us to count the number of instances of each of the elemental contact-change types for each domain movement. This in turn has led to a classification system comprising sixteen classes.

Each elemental contact-change type can be related to a model domain movement. However, although some of those classified as “pure” in Table 1 may conform to a model domain movement most domain movements comprise a mixture of contact-change types and it is probably not correct to think of these as combining the model movements. The type of contact-change may be influenced by the size and flexibility of the residues, the local structure at the interdomain region, and its proximity to the hinge axis.

By counting disconnected subgraphs in a DCG, we are able to give the number of independent regions, that is, regions comprising sets of residues between which there are no contacts in either conformation. These regions may have a different role to play in the mechanism of the domain movement.

The elemental contact-change types may relate qualitatively to the energetics of domain movements. The no contact class suggests no energy need be expended in the movement (except perhaps in the hinge bending region). The “new” type suggests energy needs to be inserted into the system or is expended. A “maintained” type suggests a strong interaction with little or no energy consumed or expended or perhaps energy being consumed or expended to strain or relieve a maintained bond. An “exchanged-partner” type may suggest a low energy barrier if a sliding movement occurs because as one interaction is weakened the other is being strengthened. An “exchanged-pair” type by contrast may indicate an energy barrier if one interaction is broken before the other one is formed in a see-saw movement. However, many domain movements are highly complex and this kind of simple interpretation will obviously not always apply. Indeed, one can imagine the exchanged-pair contact-change occurring in a way much like the sliding case if as one pair of contacts is being lost another pair of contacts is being gained such that there is no appreciable energy barrier. The work by Sinha et al. [16] suggests this mechanism with the finding that for a number of domain proteins the nonpolar buried surface area in the open state matches or slightly exceeds the nonpolar buried surface area in the closed state, especially when the domain movement is small.

For enzymes it has been shown that the type of structural change can relate to the type of reaction being catalysed [7] and it will be of interest to determine the relationship between the type of domain movement according to the classification scheme used here and molecular function.

Although we have used the DCGs to classify domain movements, they should provide, in themselves, a great deal of insight in individual cases, especially when considered by experts on the protein concerned. In essence they give a visual metaphor for the movement and its mechanism. Here we consider motifs that appear in DCGs indicating particular mechanisms.

**Figure 4:**
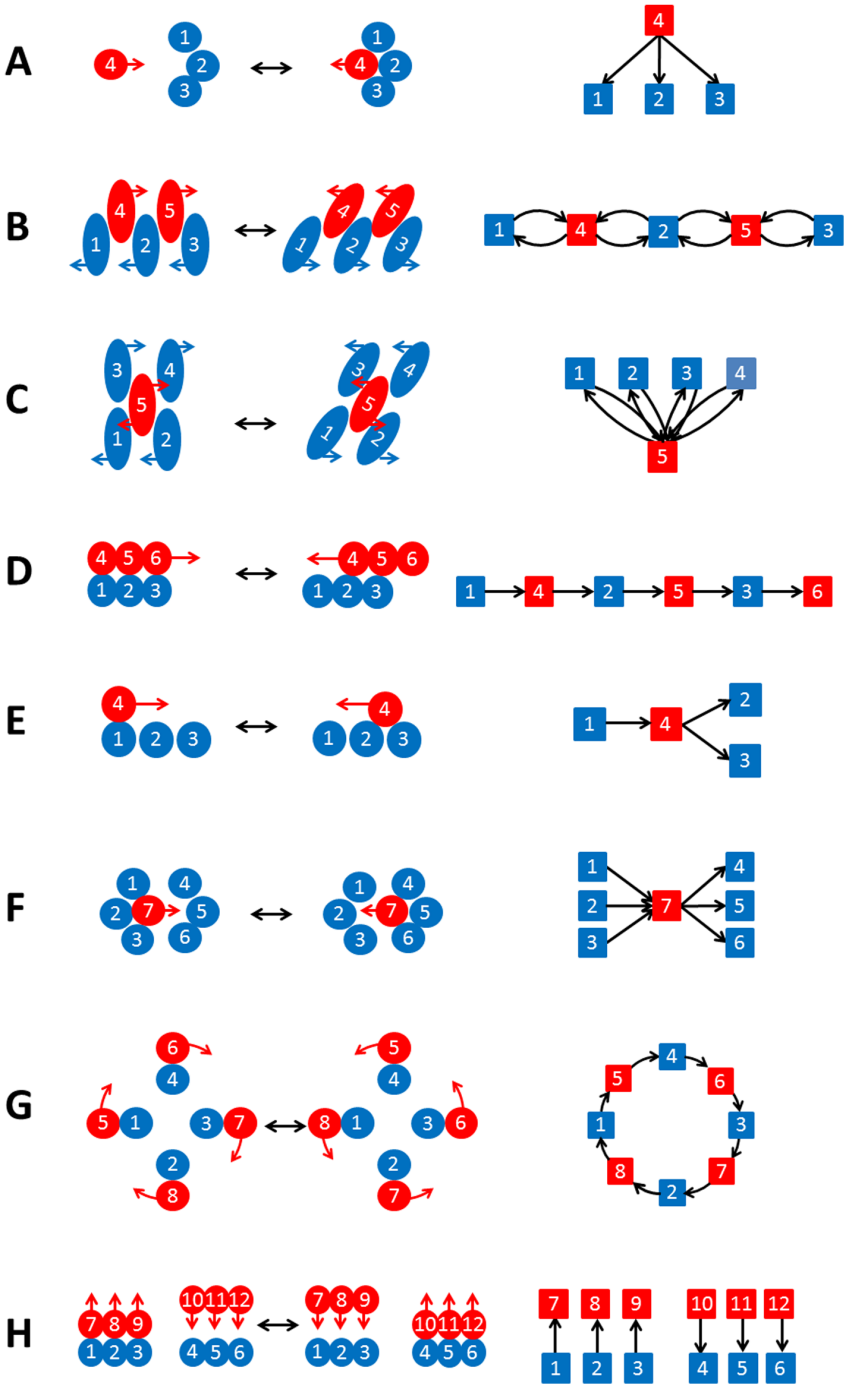
Motifs in DCG's indicating possible mechanism. Each filled circle or ellipse indicates a residue with domain A residues coloured blue and domain B residue red. Touching circles or ellipses indicate a contact. The graphs with squares and arrows are the associated DCGs. (A) “Multiple new.” A residue moves from having no contacts in one conformation to having multiple contacts in the other conformation. (B) “Linear Interlocking.” This might occur when there is a “shear” movement according to Gerstein et al. [4]. The interlocking side chains are depicted in a sequence of doubly linked nodes in the DCG suggesting strong bonds that cannot be broken. (C) “Anchoring residue.” Here a single residue maintains contact with a number of other residues from the other domain, acting possibly as an anchor. (D) “Linear slide.” Here residues slide relative to each other each making at most one contact in both conformations. The DCG depicts a set of singly connected nodes arranged linearly. (E) “Branched slide.” Here one residue makes a single contact in one conformation but two contacts in the other giving a branched DCG. (F) “Multiple-to-Multiple slide.” A residue moves from having multiple contacts with a set of residues in one conformation to multiple contacts with another set of residues in the other conformation. The DCG is clearly suggestive of this process. (G) “Closed-cycle slide.” If the domains have a twisting movement as depicted on the left the DCG will have a closed cycle. (H) “Multiple see-saw.” A see-saw movement as depicted on the left will have a DCG with edge-arrows that clearly suggest a see-saw movement.

*Multiple new*: A residue with no contact in one conformation moves into a pocket making multiple contacts in the other conformation. The associated graph is shown in Figure 4A and is a clearly recognisable motif. The domain movement in aclacinomycin 10-hydroxylase provides an example (structural pair: 1XDS, chain A; 1QZZ, chain A).

*Linear Interlocking*: A sequence of interlocking residues as depicted in Figure 1 of reference 4 for a shear movement would have a graph as shown in Figure 4B with a series of doubly linked nodes. The doubly linked nodes, give the visual metaphor of strong contacts between residues that cannot be broken. This motif is easily seen in a visual scan of a DCG. Tryptophanyl-tRNA synthetase (structural pair: 1MAU, chain A; 1I6M, chain A) provides an example.

*Anchoring residue*: A single residue maintains contact with a number of other residues during the domain movement, acting perhaps as an anchor as shown in Figure 4C. The domain movement in glucokinase provides an example (structural pair: 1Q18, chain A; 1SZ2, chain B).

*Linear slide*: A region from domain B (red in Figure 4D) sliding on a region from domain A (blue) has a graph with a series of singly linked nodes with edges all pointing in the same direction. One can think of the region from domain B sliding on the surface provided by the region of domain A with the direction of the edges indicating the direction of the movement of domain B in going from conformation 1 to conformation 2, e.g. residue 4 is moving from residue 1 to residue 2. Again the graph gives a visual metaphor for a simple sliding movement and is an easily recognised motif. The domain movement in human IGG1 FC fragment provides an example (structural pair; 1E4K, chain B; 1IWG, chain A).

*Branched slide*: If a residue in domain B makes a single contact with a residue in domain A in conformation 1 but makes contact with two residues in domain A in conformation 2 then the graph will have a branch as shown in Figure 4E. The movement in a MHC class I molecule provides an example (structural pair: 1ZT7, chain C; 1MWA, chain I).

*Multiple-to-multiple slide*: If in conformation 1 a residue in domain B makes multiple contacts with residues in domain A and moves to make multiple contacts with another region of domain A in conformation 2, the graph will be like that shown in Figure 4F. Again the graph provides a clear visual metaphor of the type of contact-change that occurs. NADH pyrophosphatase provides an example (structural pair: 1VK6, chain A; 2GB5, chain A).

*Closed-cycle slide*: If the two domains undergo a rotational motion, such that the two surfaces remain in contact, i.e. a twisting motion, and individual residues undergo a sliding movement where *every* residue makes a single contact in both conformations, then the graph will be a closed cycle as shown in Figure 4G. The associated graph clearly indicates such a rotational motion, providing a visual metaphor for the movement and an easily recognisable motif. There is always an even number of residues involved in this motif. The photosynthetic reaction centre from Thermochromatium tepidum provides an example (structural pair: 2EYT, chain A; 2EYS, chain A). As one might expect the movement in this protein is predominantly a twist (33.5% closure).

*Multiple see-saw*: If a region makes contact in conformation 1 but not in conformation 2, and a completely separate region, makes contact in conformation 2 but not in conformation 1, then the graph will look like that shown in Figure 4H. This will occur when the domains undergo a see-saw motion. The associated graph provides a strong visual metaphor for a see-saw movement. The domain movement in maltodextrin binding protein provides an example (structural pair: 1MDP, chain 2; 2OBG, chain A).

Our approach considers contacts between residues within the same subunit even if the protein functions as a multimer. Although our understanding is that domain movements in multimeric proteins involve more intrasubunit contact-changes than intersubunit contact-changes, intersubunit contact-changes need to be included in the future. The current approach was necessitated by the use of the NRDPDM which was constructed using DynDom which is only able to analyse domain movements in individual subunits. The use of a new program, DynDom3D [20], designed to analyse domain movements in multimers, will remedy this. A related issue is the absence of residue-ligand contacts in the DCGs when the ligand concerned induces the domain closure. From the viewpoint of the energetics of domain closure, the inclusion of residue-ligand contacts in the DCG would be essential, but when DCGs are used for the purpose of classifying the domain movements (e.g. whether a see-saw or a sliding-twist movement) the inclusion of these contacts should not be necessary.

Although we have limited our study to experimentally determined structures, these methods could be applied to the results of Molecular Dynamics (MD) simulation and Normal Mode Analysis (NMA). In the case of NMA a single normal mode eigenvector can be represented by two structures from which residue contacts or perhaps energy-based thresholds could be used to define the DCG. Likewise in the case of MD simulation principal component analysis gives eigenvectors from which two extreme structures can be created.

DCGs provide us with a way to identify motifs related to movements of domains. However, DCGs need not be confined to the analysis of domain movements but can be applied to any case where there are two conformations and two sets of objects e.g. subunits that have different associations in the two conformations.
